# Constraining extreme precipitation projections using past precipitation variability

**DOI:** 10.1038/s41467-022-34006-0

**Published:** 2022-11-03

**Authors:** Wenxia Zhang, Kalli Furtado, Tianjun Zhou, Peili Wu, Xiaolong Chen

**Affiliations:** 1 State Key Laboratory of Numerical Modeling for Atmospheric Sciences and Geophysical Fluid Dynamics, Institute of Atmospheric Physics, Chinese Academy of Sciences, 100029 Beijing, China; 2 Met Office, Exeter, EX1 3PB UK; 3 University of Chinese Academy of Sciences, 100049 Beijing, China

**Keywords:** Climate change, Projection and prediction

## Abstract

Projected changes of future precipitation extremes exhibit substantial uncertainties among climate models, posing grand challenges to climate actions and adaptation planning. Practical methods for narrowing the projection uncertainty remain elusive. Here, using large model ensembles, we show that the uncertainty in projections of future extratropical extreme precipitation is significantly correlated with the model representations of present-day precipitation variability. Models with weaker present-day precipitation variability tend to project larger increases in extreme precipitation occurrences under a given global warming increment. This relationship can be explained statistically using idealized distributions for precipitation. This emergent relationship provides a powerful constraint on future projections of extreme precipitation from observed present-day precipitation variability, which reduces projection uncertainty by 20–40% over extratropical regions. Because of the widespread impacts of extreme precipitation, this has not only provided useful insights into understanding uncertainties in current model projections, but is also expected to bring potential socio-economic benefits in climate change adaptation planning.

## Abstract (web summary)

This study finds that projections of future extreme precipitation can be made more reliable using a constraint from observed present-day precipitation variability, which reduces projection uncertainty by 20–40% over the extra-tropics.

## Introduction

The prospect that global warming leads to an intensified hydrological cycle and increased precipitation extremes has been supported by increasing evidence from theory, observations and model simulations^1–6^. To cope with the impacts of extreme precipitation, such as floods, disruption to ecosystems and economic growth^7^, mitigation and adaptation planning requires reliable projections of extreme precipitation. However, the current state-of-the-art climate models exhibit substantial uncertainty in the magnitudes of projected extreme precipitation changes, particularly at regional scales, despite generally agreeing on the direction of such changes^1–3^. Such uncertainty mainly arises from model differences, which lead to different model responses to identical external forcings^8^.

To achieve reliable projections and better inform decision-making, growing efforts have been devoted to understanding and constraining model uncertainty. One of the most promising approaches to reducing projection uncertainty is emergent constraint. It is based on strong statistical relationships between some observable aspects of current climate and future change across models, that can be supported by physical or mathematical reasoning^9^. For extreme precipitation projections, several emergent constraints have been proposed on large spatial scales. For tropical extreme precipitation, the projection uncertainty across models can be related to their interannual variability^10^, as well as the projected global mean precipitation change^11^. For extratropical wet regions, over large spatial aggregations, the forced response of extreme precipitation in far future is positively correlated with that in the historical period across models^12^. The robustness of an emergent constraint will be further enhanced if the statistical relationship can be supported with clear process understanding.

Despite being proposed, attempts to apply such observational constraints still face considerable challenges. Firstly, the lack of long-running, high-quality extreme precipitation observations with wide spatial coverages hinders reliable estimates of the observed variability or change. Secondly, the observed changes in extreme precipitation in past decades are affected by both external forcings and internal variability. Thirdly, the emergent relationships on extreme precipitation projections proposed so far are mostly for large spatial averages (e.g., over the tropics or extratropical wet regions as a whole); while decision-making requires regional information. Finally, our physical understanding of many proposed emergent relationships is still limited.

Here we propose a perspective to understand the model uncertainty in extreme precipitation projections, with a solid statistical basis. We show that the inter-model scatter of future changes in extreme precipitation frequency is significantly linked to model representations of present-day precipitation variability: models which have more variable precipitation in the present-day, tend to project smaller increases in extreme precipitation frequency. This emergent constraint can be understood as a statistical necessity for gamma distributions under reasonable assumptions. The constraint reduces model uncertainty in regional extreme precipitation projections in the extratropics by 20–40%.

## Results

### Establishing the emergent relationship in models

Extreme precipitation, which lies in the tails of the probability density function (PDF) of precipitation, is closely related to the width of the PDF, and hence, precipitation variability^13–15^. This has motivated us to connect model projected extreme precipitation changes with their representations of variability.

We investigate extreme precipitation changes under specific global warming levels. We use multi-model simulations in the Coupled Model Intercomparison Project Phases 5 and 6 (CMIP5 and CMIP6) under high emission scenarios to maximize the ensemble size (see Methods; Supplementary Tables 1, 2; 60 models in total). We mainly show precipitation events at synoptic timescales (consecutive 5-day precipitation, pr5d), but note that the emergent relationship holds across a range of timescales for daily- to monthly-scale events (see Methods). We focus on changes in the frequency of extreme precipitation given an extreme event threshold (moderate extremes such as the 95th percentile, R95, in the baseline; calculated using all wet and dry events), under a given global warming increment (e.g., 3 °C warming relative to present day; see Methods). This is referred to as probability ratio (${PR}$)^16^, measured by the ratio of occurrence probability in the future period and the baseline (see Methods). For precipitation variability we use the deviation of extreme events from the median state (i.e., the difference between the 95th and 50th percentile precipitation events, R95-R50)^17^. Other definitions, such as standard deviation^18,19^, are also tested but do not affect the emergent relationship (see Methods).

**Fig. 1:**
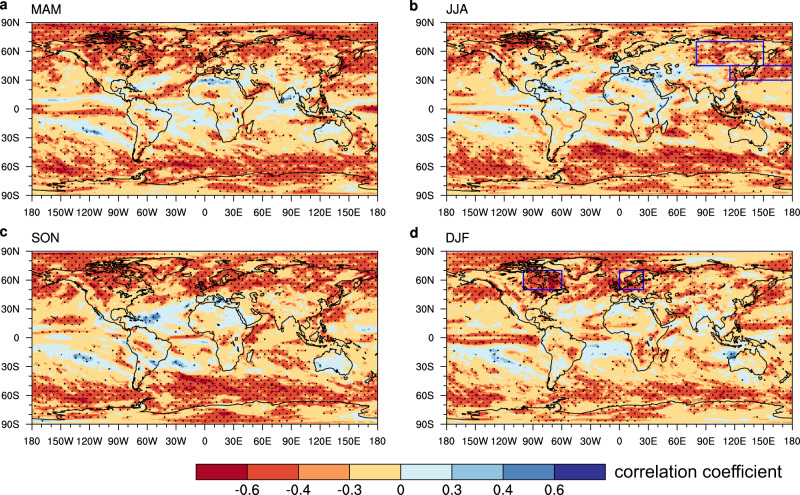
The emergent relationship in model simulations. Inter-model correlation between the present-day precipitation (pr5d) variability and the probability ratio of extreme precipitation changes under a 3 °C global warming increment in the joint ensemble of CMIP5 and CMIP6 (Coupled Model Intercomparison Project Phases 5 and 6) using Representative Concentration Pathway 8.5 (RCP8.5) and Shared Socioeconomic Pathway 5-8.5 (SSP5-8.5) scenario projections, respectively. Different seasons are considered, for March-to-May (MAM; **a**), June-to-August (JJA; **b**), September-to-November (SON; **c**) and December-to-February (DJF; **d**). Here extreme precipitation is defined as those exceeding the 95th percentile in the baseline (R95); probability ratio of extreme precipitation is measured by the ratio of occurrence probability in the future period and the baseline; precipitation variability is measured by the difference between the 95th and 50th percentile precipitation events (R95-R50). Statistically significant correlations at the 0.05 level are stippled (evaluated using a two-tailed *t-*test, assuming different models are independent).

The projected probability ratios of extreme precipitation under a given global warming increment are significantly correlated with the baseline precipitation variability across models (Fig. 1). Significant negative correlations are seen in the extratropics, exceeding −0.6 on grid point scales over most regions. This negative correlation indicates that models with weaker baseline precipitation variability project larger future change in precipitation extremes. The relationship remains steady across seasons (Fig. 1). In contrast, this relationship is loose in the tropics, which will be revisited later.

**Fig. 2:**
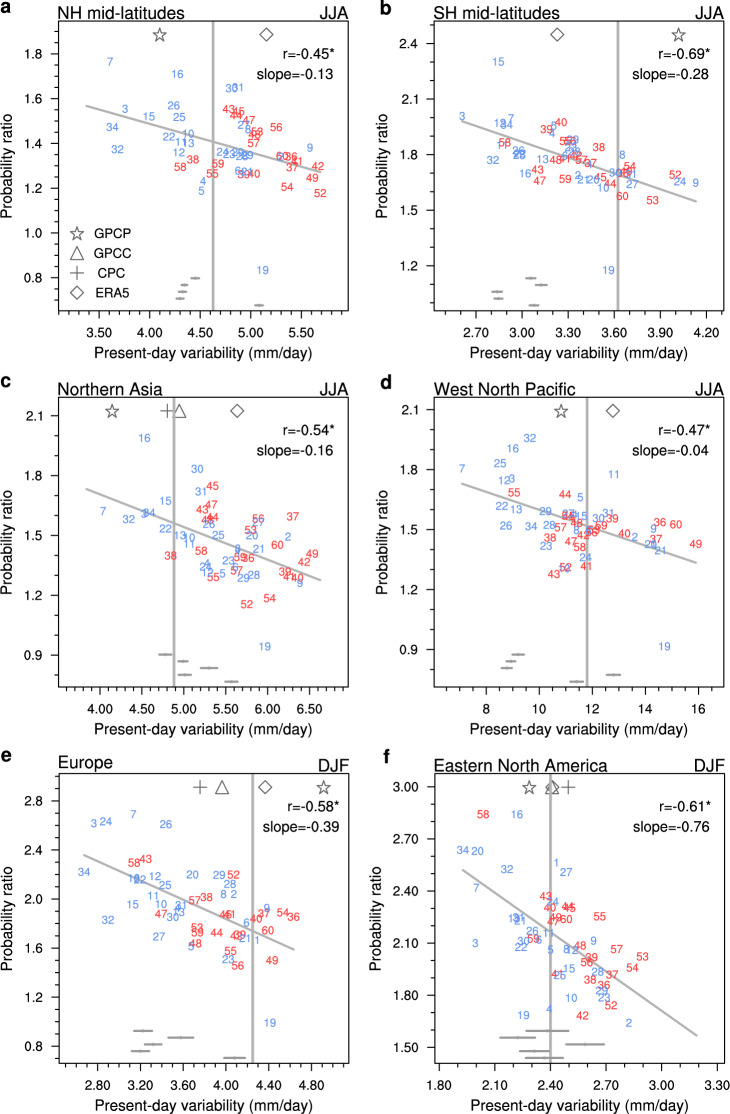
The emergent relationship over typical regions. Scatterplots of the present-day precipitation (pr5d) variability and the probability ratio of extreme precipitation (95th percentile of pr5d) changes under a 3 °C global warming increment in the joint ensemble of CMIP5 and CMIP6 (Coupled Model Intercomparison Project Phases 5 and 6). Regional cases include: Northern hemisphere mid-latitudes (45°−70°N) in June-to-August (JJA; **a**), Southern hemisphere mid-latitudes (45°−70°S) in JJA (**b**), Northern Asia in JJA (**c**), West North Pacific in JJA (**d**), Europe in December-to-February (DJF; **e**) and Eastern North America in DJF (**f**). See blue boxes in Fig. 1 for region definitions. Numbers denote individual models (blue for CMIP5 and red for CMIP6). Thin gray lines are linear fits, with correlation coefficients noted in top-right (asterisks denoting significant correlation at the 0.05 level). Markers denote present-day precipitation variability in different observations or reanalysis, with their mean values shown in vertical lines. Horizontal bars in the bottom of each panel denote the range (minimum to maximum) of internal variability in the estimation of present-day precipitation variability from 5 SMILEs (Single-Model Initial-condition Large Ensembles; see Methods).

We look further into typical regional cases where significant relationship exists, for example, the Northern hemisphere mid-latitudes (45°−70°N) in June-to-August (JJA), the Southern hemisphere mid-latitudes (45°−70°S) in JJA, Northern Asia in JJA, West North Pacific in JJA, Europe in December-to-February (DJF), and Eastern North America in DJF (see blue boxes in Fig. 1 for region definitions). The negative relationship is statistically significant over these regions across models (Fig. 2). Models with stronger present-day precipitation variability tend to project smaller increases in extreme precipitation frequency (i.e., smaller probability ratios). More than 20% of the inter-model variance in the projected probability ratios of extreme precipitation can be explained by the different representations of baseline precipitation variability over these typical regions. Over wide oceans such as the Southern hemisphere mid-latitudes where extreme precipitation is less affected by local effects, the emergent relationship is more evident with an explained variance reaching nearly 50% (Fig. 2b).

The emergent relationship identified in the joint ensemble of CMIP5 and CMIP6 could be partly affected by forcing differences in the Representative Concentration Pathway 8.5 (RCP8.5) and Shared Socioeconomic Pathway 5-8.5 (SSP5-8.5) scenarios. However, a significant emergent relationship also holds for the 1pctCO2 experiments, which use identical forcing for all models whereby atmospheric CO_2_ concentration increases by 1% per year from the pre-industrial level (Supplementary Figs. 1–2; see Methods). This agrees with our current understanding that extreme precipitation responses are more determined by the total amount of warming and model uncertainty rather than emission scenarios (Supplementary Fig. 3; see also refs. 8,20). Hence, the emergent relationship identified in scenario projections in the joint CMIP5 and CMIP6 ensemble is robust.

### Constraining extreme precipitation projections

With the identified emergent relationship, we further constrain extreme precipitation projections using observed precipitation variability. Multiple global-scale daily precipitation observations are used to account for observational uncertainty, including the Global Precipitation Climatology Project (GPCP), the Global Precipitation Climatology Centre (GPCC), National Oceanic and Atmospheric Administration (NOAA) Climate Prediction Center (CPC) unified gauge-based analysis, and the European Centre for Medium-Range Weather Forecasts (ECMWF) Reanalysis v5 (ERA5) (see Methods). Limited by the short time span of daily observations, a period of 18 years (1997–2014) is used to estimate the baseline precipitation variability for both observations and models. This estimate is not significantly affected by internal variability, as the influence of internal variability on the multi-model spread is negligible (compare the gray horizontal bars with inter-model scatters in Fig. 2; see Methods and Supplementary Table 3). Thus, the emergent relationship is dominated by model uncertainty rather than internal climate variability.

**Fig. 3:**
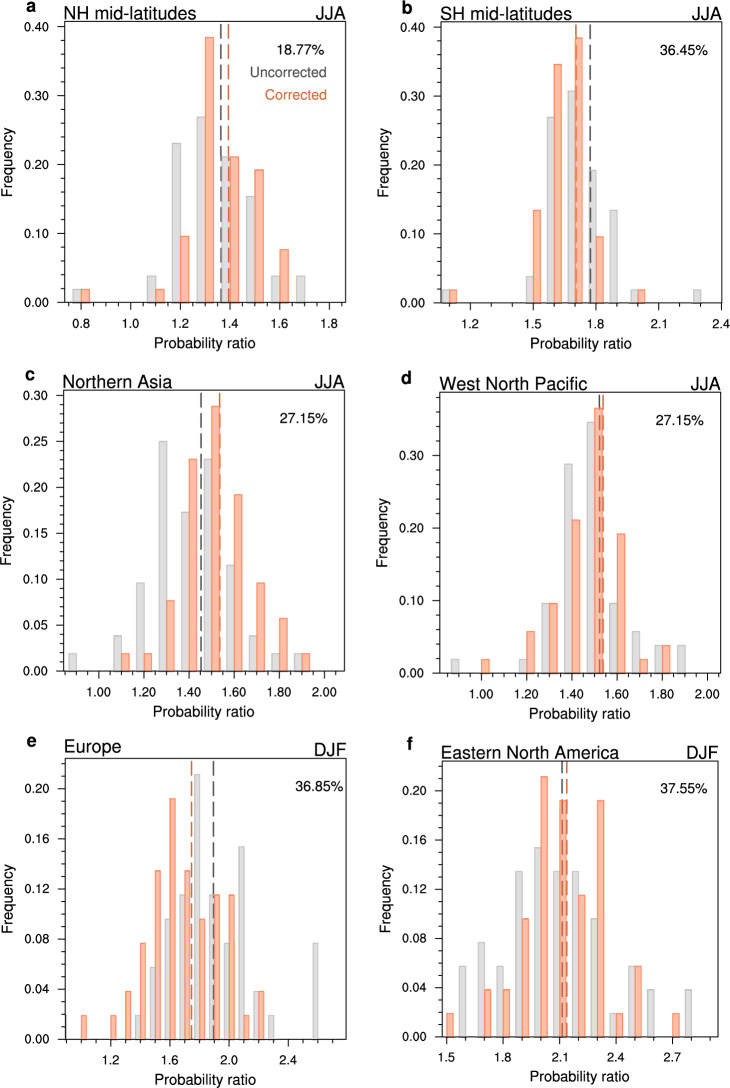
Constrained extreme precipitation projections. Unconstrained (gray) versus constrained (orange) projections of probability ratios of extreme precipitation (95th percentile of pr5d) under a 3 °C global warming increment in the joint ensemble of CMIP5 and CMIP6 (Coupled Model Intercomparison Project Phases 5 and 6). The histograms show the fraction of models with a certain probability ratio. Dashed vertical lines indicate the multi-model ensemble medians. The relative reduction in inter-model variance in projections by the constraint is noted in top-right. Regional results are shown for Northern hemisphere mid-latitudes (45°−70°N) in June-to-August (JJA; **a**), Southern hemisphere mid-latitudes (45°−70°S) in JJA (**b**), Northern Asia in JJA (**c**), West North Pacific in JJA (**d**), Europe in December-to-February (DJF; **e**) and Eastern North America in DJF (**f**).

How much uncertainty could be potentially reduced in extreme precipitation projections? We correct the model projected probability ratios of extreme precipitation by removing the linear bias due to over- or under-estimation of baseline precipitation variability^21–23^ (see Methods). Here we take the average of present-day precipitation variability estimates from multiple observations as the ‘perfect’ observation for the constraint. With the correction, the ensemble median estimates of extreme precipitation changes could either increase (i.e., more frequent) or decrease (i.e., less frequent) compared to uncorrected projections in different regions (see vertical lines in Fig. 3). This depends on whether the multi-model ensemble generally overestimates (e.g., Northern Asia in JJA; Figs. 2c, 3c) or underestimates (e.g., Europe in DJF; Figs. 2e, 3e) the baseline precipitation variability, which is region dependent (figure not shown). For example, the constrained median estimate suggests a 54% increase in extremes in Northern Asia under 3 °C warming than present day, which is 20% higher than raw projections; while for Europe, the constraint suggests a 75% increase in extremes, which is 16% lower than raw projections (Fig. 3). Meanwhile, the uncertainty range of extreme precipitation projections is consistently reduced by this constraint in all regions (Fig. 3). In the extratropics, ~20–40% of the inter-model variance in extreme precipitation projections could be potentially reduced by the observational constraint.

### Supporting the emergent constraint in a statistical framework

This emergent relationship can be interpreted in a statistical framework based on gamma distributions. We parameterize precipitation rates in the baseline climate with a gamma distribution:

1 $${f}_{k,\theta }\left(x\right),$$

where $k$ and $\theta$ are the shape and scale parameters, respectively. In response to climate warming, the precipitation distribution is transformed by simultaneously shifting and stretching the baseline distribution to obtain a new climatology:

2 $$\left(\delta,\nu {f}_{k,\theta }\right)\left(x\right):={f}_{k+\delta,\nu \theta }\left(x\right).$$

Both transformation parameters, the shift parameter $\delta$ and stretch parameter $\nu$, are closely related to changes in mean precipitation and precipitation variability (see detailed physical interpretations in Methods).

Given an extreme event threshold, the probability ratio can be calculated analytically for each transformation (see Methods). For given values of mean precipitation in the baseline climate and its fractional change in a perturbed climate, it can be shown that the probability ratio of precipitation extremes is a decreasing function of the baseline precipitation variability, on condition that the stretch parameter ($\nu$) does not differ substantially between transformations. To visualize this, Supplementary Fig. 4 shows the effect of subjecting two gamma distributions to an idealized transformation: in the example shown, the narrower baseline distribution gives a larger probability ratio of extreme precipitation. This relationship can be understood statistically as follows. Narrower distributions (i.e., those with smaller variability) have lower extreme event thresholds (e.g., the 95th percentile) than broader distributions. Therefore, given the same change in mean precipitation (similar displacement of PDFs) under warming, the lower event threshold is more easily exceeded, leading to a greater increase in extreme event occurrences for narrower distributions.

The above analysis motivates a more general claim that, for a given region, the probability ratio of extreme events is smaller in models with larger baseline variability. Implicit in this claim is an assumption that selecting a region is sufficient to uniquely determine both a baseline mean precipitation and its fractional change under a given amount of warming (the validity of this assumption will be revisited below).

**Fig. 4:**
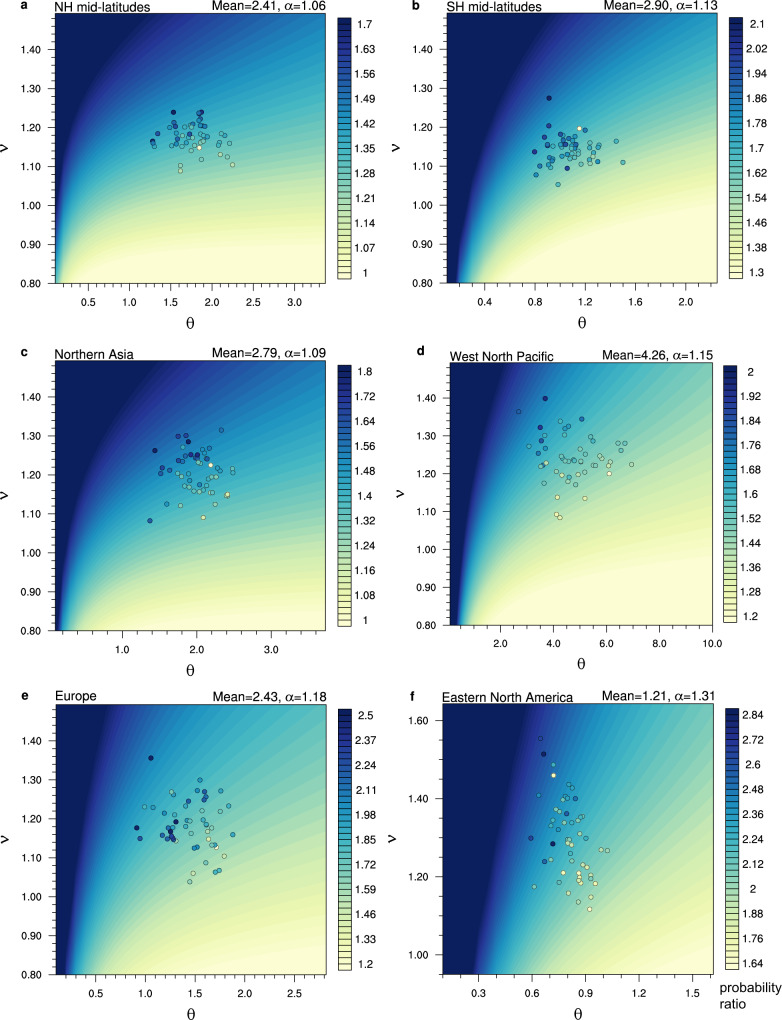
Supporting the emergent relationship in the statistical framework. Probability ratios of extreme precipitation changes as a function of gamma distribution parameters (x-axis: the scale parameter $\theta$; *y*-axis: the stretch parameter $\nu$). Background shadings are statistical calculations of probability ratios based on idealized transformations (using Eqs. 2–4; see Methods) based on multi-model medians of present-day mean precipitation (in mm/day) and its fractional change under warming ($\alpha$; noted in top-right). The dots are individual models, and their filled colors indicate model empirically estimated probability ratios (based on the frequency of occurrence of extreme events in model data). The location of model results in the parameter space is determined by fitting model data into gamma distributions (see Methods). Comparing how the background shadings and filled dots vary in the parameter space shows that the statistical argument supports the model results qualitatively. Regional results are shown for 5-day precipitation events (pr5d) for Northern hemisphere mid-latitudes (45°−70°N) in June-to-August (JJA; **a**), Southern hemisphere mid-latitudes (45°−70°S) in JJA (**b**), Northern Asia in JJA (**c**), West North Pacific in JJA (**d**), Europe in December-to-February (DJF; **e**) and Eastern North America in DJF (**f**).

This general claim is verified in a wider parameter space considering all possible transformations of distributions. Taking the selected regions as examples, we use the multi-model ensemble medians to estimate the present-day precipitation means and the fractional changes under a given warming level. The statistical calculations of probability ratios for the full range of possible transformations are shown in Fig. 4 (background shadings; see Methods). The probability ratios increase if the baseline variability (related to $\theta$) decreases or if the distributions are stretched more under warming ($\nu$ increases). This confirms our claim for the simplified case of gamma distributions.

Do the statistical argument and model results support each other? We examine how the model empirical estimates of probability ratios vary in the parameter space and qualitatively compare it with the statistical expectations (i.e., filled dots versus background shadings in Fig. 4; see Methods). Generally, the empirically estimated probability ratios from multi-models exhibit a similar pattern in the parameter space as the statistical expectation, both of which tend to increase from the bottom-right to top-left in Fig. 4. Because the modeled stretch parameters ($\nu$) vary in a relatively small range in these extratropical regions, the negative relationship between extreme precipitation probability ratios and baseline precipitation variability can emerge across models (dots in Fig. 4). Thus, the inter-model relationship can be interpreted by an idealized model based on gamma distributions, which supports the reliability and robustness of this emergent relationship.

Our idealized model also helps understand why the relationship emerges in the extratropics, but not in the tropics. This is because the assumption underlying the idealized model, i.e., that the models project similar changes in mean precipitation for a given global warming increment, holds reasonably in the extratropics but does not in the tropics (Supplementary Fig. 5). If models disagree widely on the magnitude of mean precipitation sensitivity, the probability ratios of extremes may not be dominated by baseline precipitation variability; but rather, they can be additionally affected by different displacements of the distributions. The large model uncertainty in tropical precipitation responses is dominated by the large and model-dependent dynamical changes (related to atmospheric circulation changes in response to sea surface warming patterns, changes in land-sea temperature contrast, changes in atmospheric relative humidity, etc.), which partly offset the thermodynamic contribution^24–26^. On the other hand, extratropical precipitation responses are more dominated by thermodynamic effects and are thus more robust in models.

## Discussion

Extremes, by definition, are low-likelihood high-impact events that require fine details and subtle nonlinearity from climate models to capture their occurrence and intensity. Some of those key processes currently can only be parameterized in models due to the complexity of climate system and the capacity of supercomputers. How to best utilize currently available climate model projections to assess future risks of climate extremes and to aid “safe landing” is a grand challenge to both the science community and policy makers (World Climate Research Programme Lighthouse Activity Science Plan). Given the urgent need for actionable climate science following the COP26, any reduction in uncertainties of future climate projections, climate extremes in particular, will undoubtedly bring valuable encouragement to international climate actions and economic planning.

Here we have established an inter-model relationship between extreme precipitation projections and the present-day precipitation variability. This emergent relationship can be interpreted in a simple statistical framework for gamma distributions. This statistical argument not only provides insights into understanding the projection uncertainty, but also increases the credibility of the constraint. It therefore provides an effective and physically meaningful way of constraining regional extreme precipitation projections using observed precipitation variability. The constraint not only reduces the projection uncertainty by 20–40% regionally, but also adjusts the best estimates of projections, implying altered consequences and impacts of extremes. For example, the constraint suggests a 20% greater increase in future precipitation extremes than raw projections in Northern Asia, implying an even elevated potential flood risk.

We note that the emergent constraint works well for moderate precipitation extremes but is not robust for very extreme precipitation events (e.g., those beyond the 99th percentiles; figure not shown). This can be understood statistically and physically. Statistically, the identified emergent relationship is based on the behavior of low-order moments of the entire PDF of precipitation. However, very extreme values (e.g., those beyond the 99th percentiles) lie in the far tail of the precipitation PDF and are thus less constrained by low-order moments of the precipitation PDF. Physically, extreme precipitation changes are governed by both atmospheric moisture (i.e., thermodynamic) and circulation (i.e., dynamic) changes. In particular, very extreme precipitation events are highly affected by dynamical effects (including feedbacks from latent heat release)^2,27–29^, which are model-dependent and can additionally contribute to model uncertainty in extreme precipitation responses.

The current constraint is framed in terms of probability of extreme precipitation, and is less effective for extreme precipitation intensity change (Supplementary Fig. 6 and Supplementary Discussion). Further attempts to constrain projected changes in extreme precipitation intensity, by considering, e.g., the thermodynamics and dynamics of extremes, would be very useful.

An important merit of this emergent relationship is that it holds at regional scales, and thus can be applied to different regions to make regional extreme precipitation projections more reliable. This is expected to provide actionable climate science to greatly benefit regional adaptation planning, ranging from agriculture planning and food security to flood-control systems and public safety, among many other sectors.

## Methods

### Observational and reanalysis datasets

Global-scale daily precipitation from multiple observations and reanalysis are employed to estimate precipitation variability. They include (1) the GPCP^30^ covering 1997 to present (1° × 1°), (2) the GPCC^31^ Full Data Daily Analysis covering 1982 to 2016 (1° × 1°), (3) the NOAA CPC unified Gauge-based analysis^32^ covering 1979 to present (0.5° × 0.5°), and (4) the ECMWF Reanalysis v5 (ERA5)^33^ covering 1979 to present (0.25° × 0.25°). The GPCC and CPC datasets cover only land regions. The present-day precipitation variability over 1997–2014 is used as an observational constraint (this is the common period in all the observations and model historical simulations).

### CMIP models

We use multi-model simulations of daily precipitation data from the CMIP5 (35 models; Supplementary Table 1; ref. 34) and CMIP6 (25 models; Supplementary Table 2; ref. 35) archives that are currently available. A large ensemble is required for the relationship on extreme precipitation to emerge from climate noise. The emergent relationship is established based on the joint ensemble of CMIP5 and CMIP6, using historical simulations and projections under high emission scenarios (RCP8.5 for CMIP5 and SSP5-8.5 for CMIP6). While the two emission scenarios (i.e. RCP8.5 and SSP5-8.5) have the same radiative forcing at the end of the 21st century, they differ in the pathway.

To test the robustness of the emergent relationship against scenario difference, the emergent relationship is further examined under identical external forcings for both CMIP5 and CMIP6 models, using the 1pctCO2 experiment, where the atmospheric CO_2_ concentration increases by 1% per year from the pre-industrial level. Results show that the inter-model scatter of extreme precipitation responses, and hence, the emergent relationship, is dominated by model uncertainty rather than forcing/scenario difference.

### Changes in extreme precipitation

Precipitation events on a range of timescales from daily to monthly are considered, namely, prNd (*N* = 1, 5, 30), by applying a running average to daily precipitation over N days. The precipitation indices, prNd, is first computed on model native grids and then regridded to a common 1° × 1° grid boxes. Since rainy season varies between regions, we consider precipitation events in different seasons, namely, March to May (MAM), June to August (JJA), September to November (SON), and December to February (DJF). We note that the emergent relationship holds across the different timescales for 1-day, 5-day and 30-day precipitation events, we only show the results of pr5d in the main manuscript for brevity (see Supplementary Figs. 7, 8 for the pr1d results).

Extreme precipitation events are defined as those exceeding a high percentile (say, the 95th percentile, R95) in the present-day baseline (1997–2014). Alternative event thresholds including R90, R98 and R99 are also tested which do not affect the emergent relationship (see Supplementary Fig. 9 for example results of R99). We note that the emergent constraint works well for moderate precipitation extremes but is not robust for very extreme precipitation events (e.g., those beyond the 99th percentiles; see the Discussions section). In this study, all days (including wet and dry days) are used to compute the percentiles. It is preferable to calculate extreme precipitation using all days rather than only wet days when comparing models with observations, because climate models have a common bias to simulate too-frequent precipitation events, which affects percentiles calculated over only wet days^2^.

We investigate changes in the frequency of extreme precipitation under future warming, referred to as probability ratio^16^ (${PR}$). It is measured as the ratio of occurrence probability of an extreme event (with a given threshold) in a warmer climate and that in the baseline.

We consider warmer climates with a specific (such as 2 °C, 3 °C or 4 °C) global warming increment relative to present day for each model; 20-year segments are used for all the future periods. While the emergent relationship remains significant when different warming levels are considered (figure not shown), there is a trade-off between higher and lower global warming levels. Under higher global warming levels, extreme precipitation changes are less affected by internal variability and thus exhibit a larger signal-to-noise ratio, the inter-model relationship can clearly emerge from climate noise. However, fewer models can reach a higher global warming level (e.g., 25 out of 60 CMIP5/CMIP6 models reach a 4 °C warming under high emission scenarios), resulting in a smaller sample size. Thus, as a trade-off between the signal-to-noise ratio of extreme precipitation changes and the sample size, we mainly show a 3 °C global warming level relative to present day as the warmer condition. A total of 52 models reaching a 3 °C warming is used to investigate the emergent relationship.

### Estimation of precipitation variability

Precipitation variability indicates the range that precipitation events can vary in time. It can be represented by the standard deviation of precipitation timeseries^18,19^. Alternatively, it can be represented by the extent to which extreme events deviate from the normal state, such as the difference between the 95th or 98th percentile precipitation event and the 50th percentile event (i.e., R95-R50 or R98-R50; ref. 17). We have confirmed that the emergent relationship is insensitive to the different estimations of precipitation variability. For brevity, precipitation variability estimated as R95-R50 is shown in the main manuscript (see Supplementary Fig. 10 for example results when precipitation variability is measured by standard deviation).

We emphasize that the emergent relationship is robust and insensitive to the timescales of precipitation events, extreme event thresholds, and methods of estimating precipitation variability. This increases the robustness of the emergent relationship.

### Supporting the emergent relationship in the statistical framework

We interpret the emergent relationship in a statistical framework for gamma distributions. The claim is, for a given location, given the same displacement in gamma distributions between the control and perturbed warmer climate, models with larger baseline precipitation variability (i.e., wider PDF) yield smaller probability ratios of extreme precipitation changes. In the statistical framework, it is required that different models project the same displacement in precipitation PDFs under warming. This is approximated by requiring models projecting the same change in mean precipitation. To meet that, we assume that for a given location, (1) models have the same mean precipitation in the current climate state, and (2) they project the same fractional change in mean precipitation given the same amount of warming. This is reasonable as current global climate models have basic ability in simulating mean precipitation climatology^36^.

To formulate, given a gamma distribution for precipitation in the control climate:

1 $${f}_{k,\theta }\left(x\right),$$

where $k$ and $\theta$ are parameters. The scale parameter, $\theta$, is an indicator of how rapidly precipitation frequency declines as precipitation intensity increases, for relatively heavy precipitation events. The shape parameter $k$ determines how rapidly precipitation frequency decays or grows for precipitation events with intensities much lower than $\theta$. A larger $k$ leads to a less skewed distribution and shifts the distribution to the right. The mean of the gamma distribution is $k\theta$, and the variance is $k{\theta }^{2}$.

In a perturbed warmer climate, where the precipitation distribution is shifted and stretched simultaneously, under transformations of the form:

2 $$\left(\delta,\nu {f}_{k,\theta }\right)\left(x\right):={f}_{k+\delta,\nu \theta }\left(x\right),$$

where $\delta$ and $\nu$ are transformation parameters, both of which are closely related to changes in mean precipitation and precipitation variability. The shift parameter, $\delta$, is the fractional change in mean precipitation at constant scale parameter (i.e. $\nu=1$), i.e., the precipitation change that occurs if the distribution changes shape without altering the slope of the PDF tail. Similarly, the stretch parameter, $\nu$, is related to fractional change in precipitation variability at constant shape parameter (i.e. $\delta=0$). For the new gamma distribution, the mean is $\left(k+\delta \right)\nu \theta$, and the variance is $\left(k+\delta \right){\left(\nu \theta \right)}^{2}$.

Here we mainly consider regions with an increasing mean precipitation under warming, as these regions are generally expected to experience increasing extreme precipitation and associated impacts, thus requiring reliable projections. These regions cover widely over the globe except for some subtropical subsidence regions^37^ (Supplementary Fig. 11).

We assume that both the baseline mean precipitation ($k\theta$) and its fractional change ($\alpha$) are constant for a given location. The latter can be expressed as:

3 $$\alpha :=\left(1+\frac{\delta }{k}\right)\nu,$$

where $\alpha$ is constant and larger than 1. We also note that, for a region, with the baseline mean precipitation ($k\theta$) being constant, the baseline variability or variance ($k{\theta }^{2}$) is solely determined by the scale parameter $\theta$. For each transformation, ${S}_{\delta,\nu }$, define the probability ratio (${PR}$) for an extreme event threshold $r$:

4 $${{PR}}_{\delta,\nu }\left(r;k,\theta \right){:}{=}\frac{{\int}_{r}^{{{\infty }}}{f}_{k+\delta,\nu \theta }\left(x\right){dx}}{{\int }_{r}^{{{\infty }}}{f}_{k,\theta }\left(x\right){dx}}.$$

Then the claim is, for a given location where $k\theta$ is constant, given a fractional increase in the mean ($\alpha\, > \,1$), for all transformations with $\delta,\nu$ such that $\left(1+\frac{\delta }{k}\right)\nu=\alpha$, the probability ratios of an extreme event (with threshold $r$), ${{PR}}_{\delta,\nu }\left(r\right)$, are decreasing functions of the baseline variability (related to $\theta$).

We verify this claim by analytically estimating probability ratios for the full range of possible transformations with different shift ($\delta$) and stretch ($\nu$) parameters, based on Eqs. (2–4) (see background shadings in Fig. 4 for regional examples). The statistical estimates of probability ratios increase as baseline variability ($\theta$) decreases, on condition that the stretch parameter ($\nu$) does not vary widely between transformations (Fig. 4). This confirms our claim.

To interpret the inter-model relationship in the statistical framework, here we examine how the model empirical estimates of probability ratios vary in the parameter space and qualitatively compare it with the statistical expectations (cf. filled dots and background shadings in Fig. 4). In Fig. 4, the dots denote individual models and their filled colors indicate the model empirically estimated probability ratios (based on the frequency of occurrence of extreme events in model data). To determine their location in the parameter space, we need to estimate the parameters of precipitation PDF for each model. This is achieved by fitting the model present-day and future precipitation time series to gamma distributions (using Maximum Likelihood), and then estimating the distribution parameters based on Eqs. (2–3). The distribution parameters are first estimated on each grid cell and then the area-weighted regional averages are derived, which are then used to determine the location for each model in the parameter space in Fig. 4. As a necessity for fitting the gamma distributions^38^, only precipitating events exceeding 0.1 mm/day are used for the fit^39,40^. We note that the gamma distribution fit is employed only to estimate the parameters of precipitation PDF in models (as a simplification of the actual change), based on which we interpret the inter-model relationship between the PDF parameters and extreme precipitation probability ratio—such interpretation can only be qualitative.

Comparing how the background shadings and filled dots in Fig. 4 vary in the parameter space shows that the statistical argument supports the model results. Generally, the statistical calculations and model-derived empirical estimates of probability ratios vary in the same direction in the parameter space, both increasing as the baseline variability ($\theta$) decreases and as the stretch parameter ($\nu$) increases (Fig. 4). As the modeled stretch parameters ($\nu$) vary in a relatively small range in these extratropical regions, the negative relationship between extreme precipitation probability ratios and baseline precipitation variability can emerge across models (dots in Fig. 4). Thus, the emergent relationship in models is well supported by the statistical argument. Note that we should not expect the probability ratios from the statistical calculations and model direct empirical estimates to be identical, because the precipitation distribution changes in climate models are far more complicated than the simplified transformations and the statistical calculations are based purely on the multi-model median parameters.

Because the probability ratio of extreme precipitation is a function of the baseline variability (related to $\theta$) and the stretch parameter ($\nu$), the inter-model relationship between the probability ratio and baseline variability only emerges if the inter-model spread of the stretch parameter is not too wide (Fig. 4). To assess the validity of this condition, we need to know what determines the inter-model spread of the stretch parameter. Based on the above statistical argument, the stretch parameter ($\nu$) is proportional to the fractional change in precipitation variance, given the fractional change in mean precipitation ($\alpha$) being constant:

5 $$\frac{{\sigma }^{2}(P1)}{{\sigma }^{2}(P0)}=\frac{(k+\delta ){\nu }^{2}{\theta }^{2}}{k{\theta }^{2}}=\alpha \cdot \nu,$$

where $P0$ and $P1$ denote precipitation in the baseline and perturbed climate, respectively, and ${\sigma }^{2}$ denotes variance. It has been demonstrated that the standard deviation of precipitation is proportional to mean atmospheric humidity and the standard deviation of vertical motion (see ref. 19 and their Eq. 12). This means that the stretch parameter ($\nu$) is affected by a thermodynamic factor (related to change in mean atmospheric humidity) and a dynamic factor (related to change in circulation variance). The circulation change has been recognized to be far more uncertain than the humidity change in model projections^41^. Thus, the inter-model spread in the stretch parameter is dominated by that in the change of circulation variance. Hence, the validity of the above condition on the constraint (that the inter-model spread of stretch parameter is not too wide) depends on the projection uncertainty of circulation variance change. The latter is itself an interesting question that deserves dedicated research in the future.

The above statistical argument is established for regions where mean precipitation increases. Now we discuss how it applies to regions with mean precipitation decreases. First, for cases where both the mean and extreme precipitation decrease under warming, the results can be inferred by ‘inverting’ the heuristic argument above. See again Supplementary Fig. 4 for illustration. We have demonstrated that, across models, for regions where both the mean and extreme precipitation increase (i.e., from climate state 0 to climate state 1), precipitation variability is negatively correlated with the probability ratio of extreme precipitation:

6 $${PR}={Prob}1/{Prob}0,$$

where ${Prob}0$ and ${Prob}1$ are extreme event probability in respective climate states. Thus, in inverse cases where both the mean and extreme precipitation decrease (i.e., from climate state 1 to climate state 0), precipitation variability is expected to be positively related to probability ratio (see Supplementary Fig. 4 for illustration):

7 $${PR}^{\prime}={Prob}0/{Prob}1,$$

where ${PR}^{\prime}$ denotes extreme precipitation probability ratio in inverse cases. Indeed, in some subtropical subsidence regions (where both mean and extreme precipitation decrease; Supplementary Fig. 11), there is positive correlation from model results as seen in Fig. 1, although significant correlations are limited and patchy.

Second, for cases where mean precipitation decreases but extreme precipitation increases (i.e., hatched regions in Supplementary Fig. 11), there is no significant correlation. As these are mainly transition zones between regions getting wetter and drier, the mean precipitation change is close to zero with low model agreement in the sign (Supplementary Fig. 11). This means the displacement of precipitation PDF is small and the direction of displacement is uncertain. As a result, the small PDF displacement may not be the main factor of extreme precipitation changes; instead, changes in PDF tails could play important roles in inducing extreme precipitation changes and their inter-model differences. This is inconsistent with the proposed argument, thus no significant relationship is expected in these regions.

### Confirming the emergent relationship using the 1pctCO2 experiment

We confirm the emergent relationship under identical external forcings for all models using the 1pctCO2 experiment. Here the baseline is defined as the first 20 years in the 1pctCO2 experiments, and the warming conditions are defined at a 3 °C global warming level (using 20-year periods) under the 1pctCO2 forcing relative to the baseline for each model. Despite with a smaller multi-model ensemble (due to the availability of model data; 39 models in 1pctCO2 experiments versus 52 models in scenario projections, under a 3 °C warming increment), the significant negative correlation between the baseline precipitation variability and probability ratios of extreme precipitation remains consistent under the 1pctCO2 forcing as that in scenario projections (Supplementary Figs. 1, 2). This confirms the robustness of the emergent relationship.

### Influence of internal variability on the estimation of precipitation variability

Limited by the availability of global-scale daily precipitation observations, a short period of 18 years (1997–2014) is used to estimate the baseline precipitation variability. To explore the potential influence of internal variability on the estimation of precipitation variability, five Single-Model Initial-condition Large Ensembles (SMILEs) produced by the US CLIVAR Working Group on Large Ensembles are employed^42^. The 5 SMILEs providing daily precipitation output are CESM1, CanESM2, CSIRO-MK3.6, GFDL-CM3, and EC-Earth (Supplementary Table 3). Daily precipitation from historical simulations and RCP8.5 projections are used.

Within each SMILE, all the realizations are driven by the same external forcings and differ only in initial conditions. Thus, the inter-member difference within a SMILE represents internal variability.

### Correction of model projections

In the framework of emergent constraint, once the inter-model relationship between future climate change *Y* and current climate *X* has been established, a simple linear approximation between *Y* and *X* can be obtained based on multi-model ensembles^21–23^:

8 $$Y={aX}+b,$$

where *a* is the regression coefficient and *b* is the regression constant (evaluated via Ordinary Least Squares). In this study, *Y* is the probability ratio of extreme precipitation changes given an event threshold, and *X* is the baseline precipitation variability. Where this relationship is statistically significant, the projection *Y* can be calibrated based on this inter-model relationship and the observed baseline precipitation variability ${X}_{{obs}}$. Firstly, the bias in the simulated baseline precipitation variability, ${X}_{{bias}}$, is obtained for each model:

9 $${X}_{{bias}}=X-{X}_{{obs}}.$$

We then estimate the projection errors, ${Y}_{{bias}}$, induced by ${X}_{{bias}}$ based on the identified relationship, for each model:

10 $${Y}_{{bias}}={{aX}}_{{bias}}=a\left(X-{X}_{{obs}}\right).$$

Finally, the corrected projections, ${Y}_{{corrected}}$, are obtained by removing this linear bias for each model:

11 $${Y}_{{corrected}}=Y-{Y}_{{bias}}.$$

## Supplementary information

Supplementary Information

## Data Availability

Daily precipitation observations or reanalysis can be acquired from: (1) https://www.ncdc.noaa.gov/cdr/atmospheric/precipitation-gpcp-daily for GPCP, (2) https://opendata.dwd.de/climate_environment/GPCC/html/download_gate.html for GPCC, (3) https://psl.noaa.gov/data/gridded/data.cpc.globalprecip.html for CPC, and (4) https://www.ecmwf.int/en/forecasts/datasets/reanalysis-datasets/era5 for ERA5. CMIP5 and CMIP6 data can be acquired from https://esgf-node.llnl.gov/projects/esgf-llnl/. Single-Model Initial-condition Large Ensembles can be acquired from https://www.cesm.ucar.edu/projects/community-projects/MMLEA/. Key processed data have been deposited at 10.7910/DVN/UZC5YL. Map data in Fig. 1 and Supplementary Figs. 1, 5–7, and 11 is from the NCAR Command Language (Version 6.6.2; 2019) [Software] (Boulder, Colorado: UCAR/NCAR/CISL/TDD. 10.5065/D6WD3XH5).

## Code availability

Key codes can be accessed from 10.7910/DVN/UZC5YL.
