# Supplementary material for: Constraining extreme precipitation projections using past precipitation variability
Source: Nat Commun. 2022 Nov 3;13:6319. doi: 10.1038/s41467-022-34006-0 (PMC9633619; doi:10.1038/s41467-022-34006-0)
Supplement: Supplementary file 1 — Supplementary Information [file 41467_2022_34006_MOESM1_ESM.pdf]

Supplementary Information

Constraining extreme precipitation projections using past precipitation variability

Wenxia Zhang[1], Kalli Furtado[2], Tianjun Zhou[1,3*], Peili Wu[2], Xiaolong Chen[1]

1 State Key Laboratory of Numerical Modeling for Atmospheric Sciences and Geophysical Fluid Dynamics, Institute of Atmospheric Physics, Chinese Academy of Sciences, Beijing, 100029, China.

2 Met Office, Exeter, EX1 3PB, UK.

3 University of Chinese Academy of Sciences, Beijing, 100049, China.

The file contains:

- Supplementary Discussion
- Supplementary Figures 1-11
- Supplementary Tables 1-3

[*] Corresponding author: Tianjun Zhou (zhoutj@lasg.iap.ac.cn)

Supplementary Discussion. Testing the constraint for extreme precipitation intensity change

The current constraint is framed in terms of probability of extreme precipitation, here we test if it also works for extreme precipitation intensity change. The inter-model correlation between the present-day precipitation variability and extreme precipitation intensity change (in mm/day) is shown in Supplementary Fig. 6. The results are overall insensitive to different timescales of precipitation events and different measures of precipitation variability, thus only 5-day precipitation events (pr5d) are shown for brevity. No significant and systematic correlation exists in the mid-to-high latitudes, although there are patchy areas of statistically significant positive (negative) correlations in the tropical wet (subtropical dry) regions, which weaken for heavier extremes (Supplementary Fig. 6).

Therefore, present-day precipitation variability plays a lesser role in the inter-model differences of extreme precipitation intensity change, and is thus not a powerful constraint. As extreme precipitation intensity is directly governed by thermodynamic (related to atmospheric humidity changes) and dynamic (related to circulation changes) processes, it may be more promising to connect projected changes in extreme precipitation intensity (and further, their model uncertainty) with key thermodynamics and dynamics. This is expected to improve the understanding of the projection uncertainty of extreme precipitation intensity and deserves dedicated research.

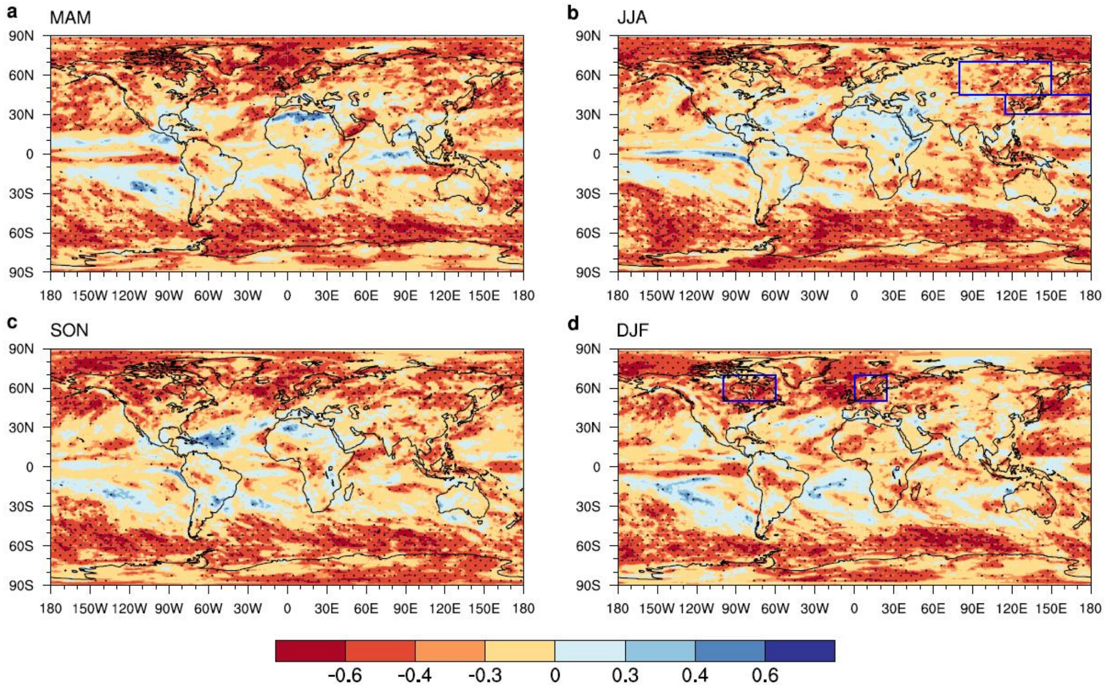

Supplementary Fig. 1 The emergent relationship in model simulations. Inter-model correlation between the baseline precipitation (pr5d) variability and the probability ratio of extreme precipitation changes under a 3°C warming level under 1pctCO2 forcing in the joint ensemble of CMIP5 and CMIP6, for MAM (**a**), JJA (**b**), SON (**c**) and DJF (**d**). The baseline is defined as the first 20 years in the 1pctCO2 experiments, and the warming conditions are defined at a 3°C global warming level (using 20-year periods) under the 1pctCO2 forcing relative to the baseline for each model. Here extreme precipitation is defined as those exceeding the 95th percentile in the baseline (R95); probability ratio of extreme precipitation is measured by the ratio of occurrence probability in the future period and the baseline; precipitation variability is measured by the difference between the 95th and 50th percentile precipitation events (R95-R50). Statistically significant correlations at the 0.05 level are stippled.

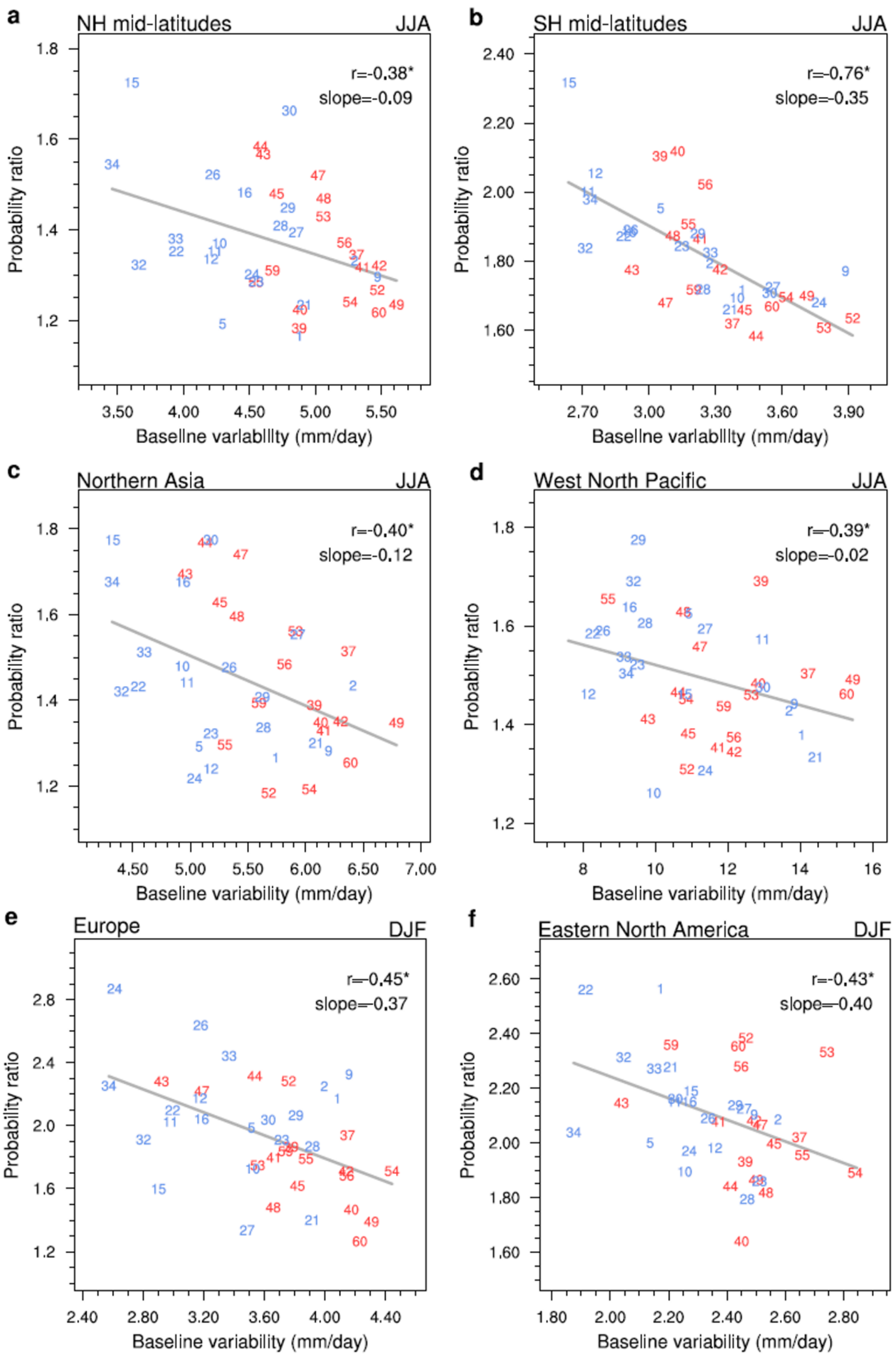

Supplementary Fig. 2 The emergent relationship over typical regions. Scatterplots of the baseline precipitation (pr5d) variability and the probability ratio of extreme

precipitation (95th percentile of pr5d) changes under a 3°C global warming increment under 1pctCO2 forcing in the joint ensemble of CMIP5 and CMIP6. Regional results are shown for Northern hemisphere mid-latitudes (45°-70°N) in JJA (**a**), Southern hemisphere mid-latitudes (45°-70°S) in JJA (**b**), Northern Asia in JJA (**c**), West North Pacific in JJA (**d**), Europe in DJF (**e**) and Eastern North America in DJF (**f**). See Fig.1 for region definitions. Numbers denote individual models (blue for CMIP5 and red for CMIP6). Thin grey lines are linear fits, with correlation coefficients noted in top-right (asterisks denoting significant correlation at the 0.05 level).

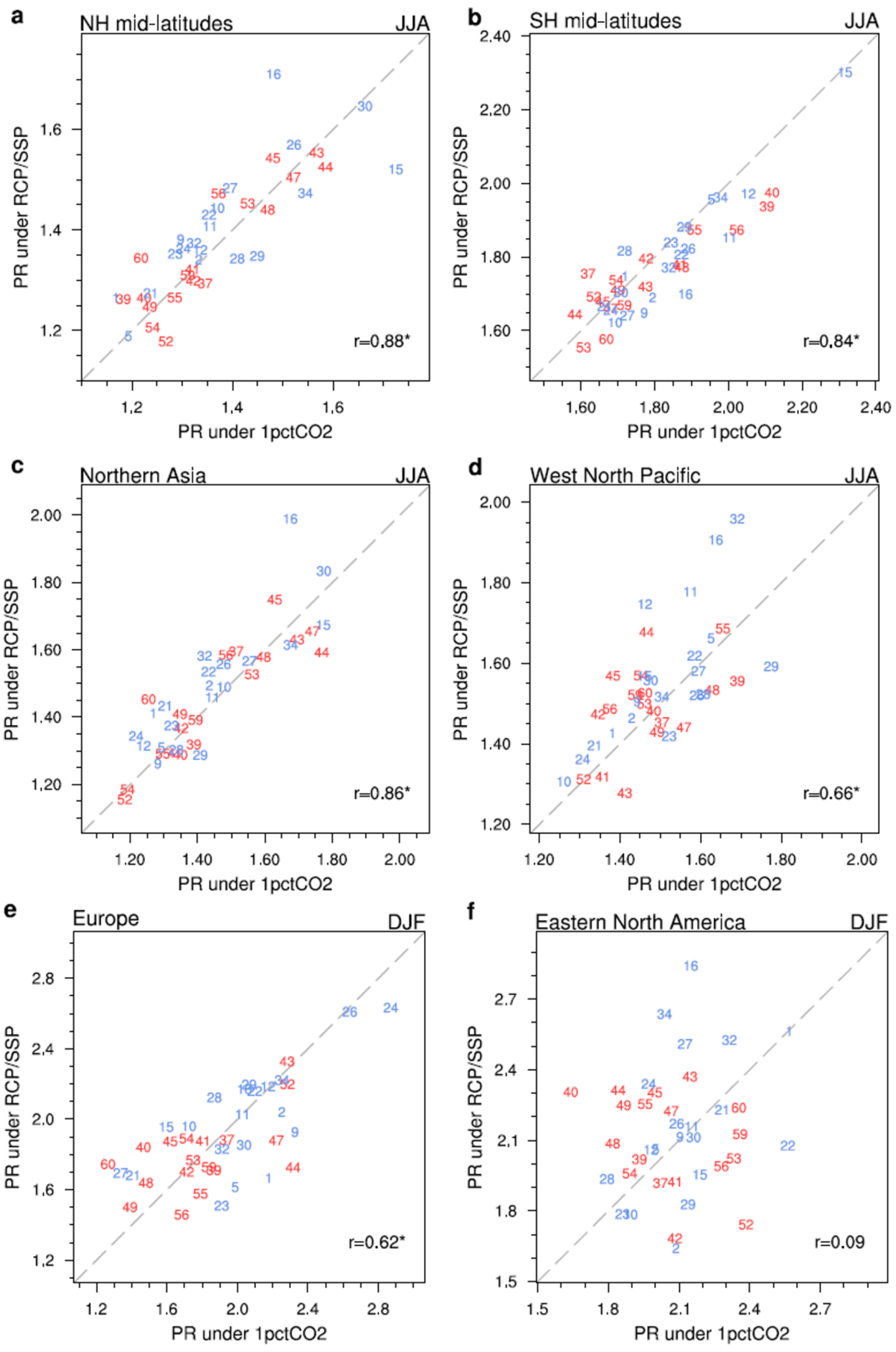

Supplementary Fig. 3 Comparison of extreme precipitation probability ratio under scenario projections and 1pctCO2 experiment. Scatterplots of the probability

ratio (PR) of extreme precipitation (95th percentile of pr5d) changes under a 3°C warming increment under 1pctCO2 forcing (x-axis) versus that under scenario projections (RCP8.5 for CMIP5 and SSP5-8.5 for CMIP6) (y-axis). Regional results are shown for Northern hemisphere mid-latitudes (45°-70°N) in JJA (**a**), Southern hemisphere mid-latitudes (45°-70°S) in JJA (**b**), Northern Asia in JJA (**c**), West North Pacific in JJA (**d**), Europe in DJF (**e**) and Eastern North America in DJF (**f**). See Fig.1 for region definitions. Numbers denote individual models (blue for CMIP5 and red for CMIP6). Correlation coefficients are noted in bottom-right (asterisks denoting significant correlation at the 0.05 level). Dashed lines indicate y=x. In most cases, the multi-model responses in extreme precipitation under 1pctCO2 and RCP8.5/SSP5-8.5 forcings spread along the diagonal line of y=x, with a significant inter-model correlation, suggesting that the inter-model scatter of extreme precipitation responses is dominated by model uncertainty, with a small influence from forcing difference.

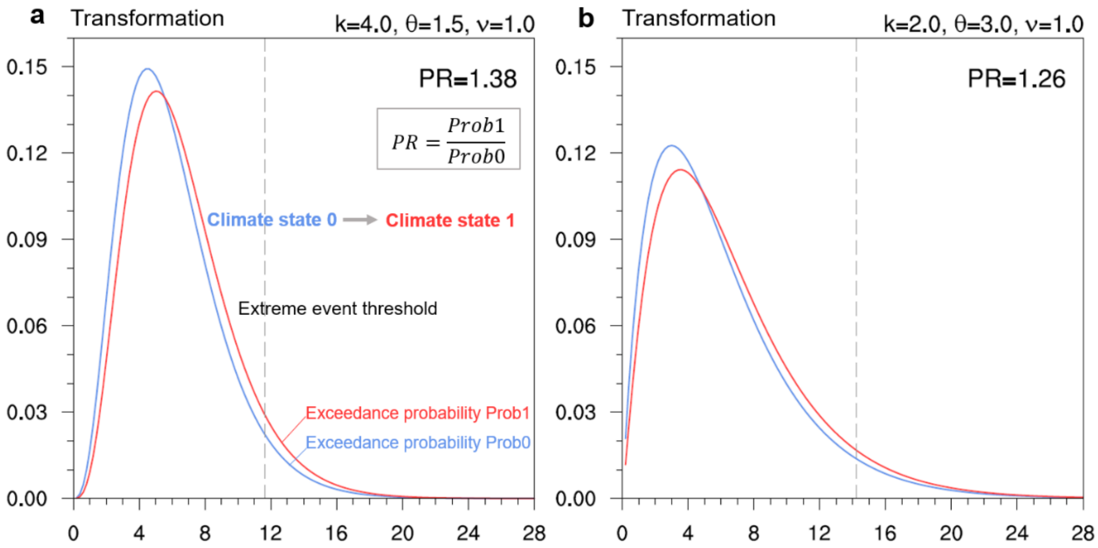

Supplementary Fig. 4 Illustration of the emergent relationship. Two illustrating examples of gamma distributions with the same mean state ($k\theta = 6$) but different variability (different scale parameter, θ) in the control climate (climate state 0; blue curves). In a perturbed climate (climate state 1; red curves), given the same fractional change in mean precipitation (i.e., α=1.09 in Eq. (3)), there are various possibilities to transform the precipitation PDF with different combinations of shift (δ) and stretch (ν) parameters, under the constraint of Eq. (3). On condition that the PDFs are stretched to a similar extent (i.e., similar ν; here $\nu = 1$ in both cases), the narrower distribution in (**a**) (with smaller θ) yields a larger probability ratio (PR) of extreme precipitation changes than the broader distribution in (**b**). Here extreme event threshold is defined as the 95th percentile in the baseline (dashed vertical line).

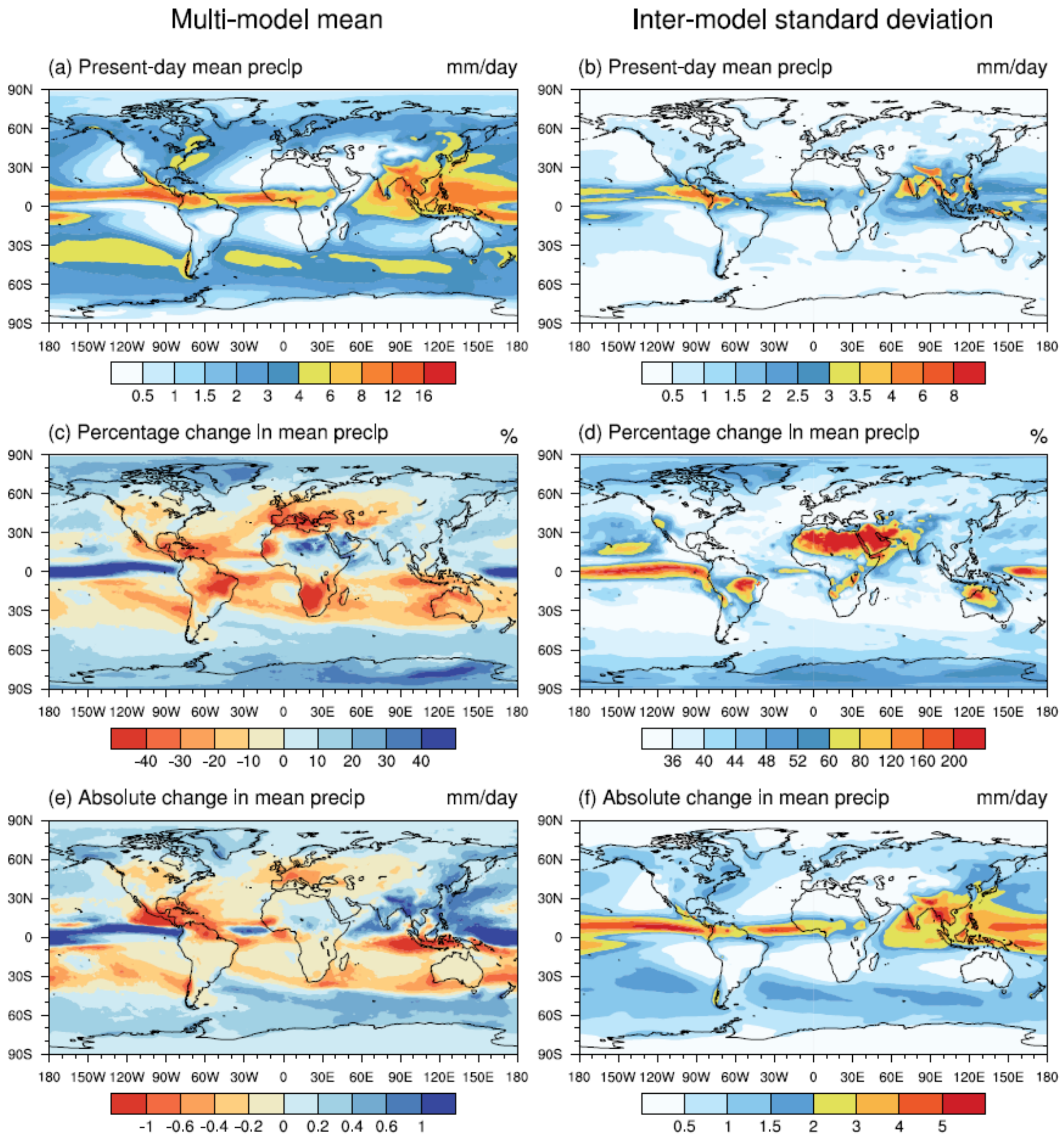

Supplementary Fig. 5 Projected changes in mean precipitation. a, Multi-model ensemble mean present-day mean precipitation (in mm/day). **b,** Inter-model standard deviation of present-day mean precipitation (in mm/day). **c,** Multi-model ensemble mean projected percentage changes in mean precipitation under a 3°C warming increment (RCP8.5 scenario for CMIP5 and SSP5-8.5 for CMIP6 models) (in % relative to present-day climatology). **d,** Inter-model standard deviation of projected mean precipitation changes (in %). **e-f,** Same as **c-d**, but for projected absolute changes in mean precipitation (in mm/day). Results show that multi-models agree better in the

projected mean precipitation changes (in absolute terms) in the extratropics with smaller inter-model standard deviation than in the tropics (Supplementary Fig. 5f). This is contributed by both the higher model consistency in the simulated baseline mean precipitation and the fractional change in mean precipitation in the extratropics (Supplementary Fig. 5b,d), which agree with the assumptions in the statistical framework. These results are consistent across seasons; only the June-to-August season is shown here.

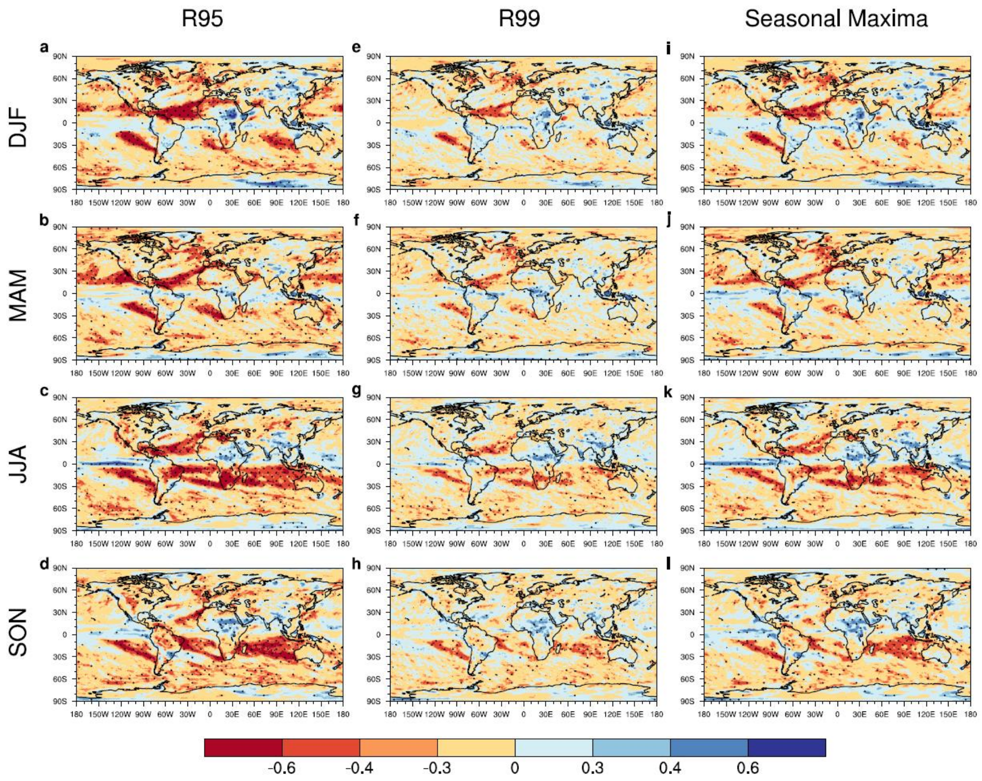

Supplementary Fig. 6 Inter-model correlation between the present-day precipitation variability and extreme precipitation intensity change. Inter-model correlation between the present-day precipitation variability and extreme precipitation intensity change (in mm/day) under a 3°C global warming increment in the joint ensemble of CMIP5 and CMIP6 using RCP8.5 and SSP5-8.5 scenario projections, respectively. The results for 5-day precipitation events (pr5d) are shown. Different extremes are considered, including the 95th percentile (R95; **a-d**), the 99th percentile (R99; **e-h**) and seasonal maximum precipitation event (**i-l**). Different seasons are shown, for December-to-February (DJF; the 1st row), March-to-May (MAM; the 2nd row), June-to-August (JJA; the 3rd row), and September-to-November (SON; the 4th row). Here precipitation variability is measured by the difference between the 95th and 50th percentile precipitation events (R95-R50). Statistically significant correlations at the 0.05 level are stippled.

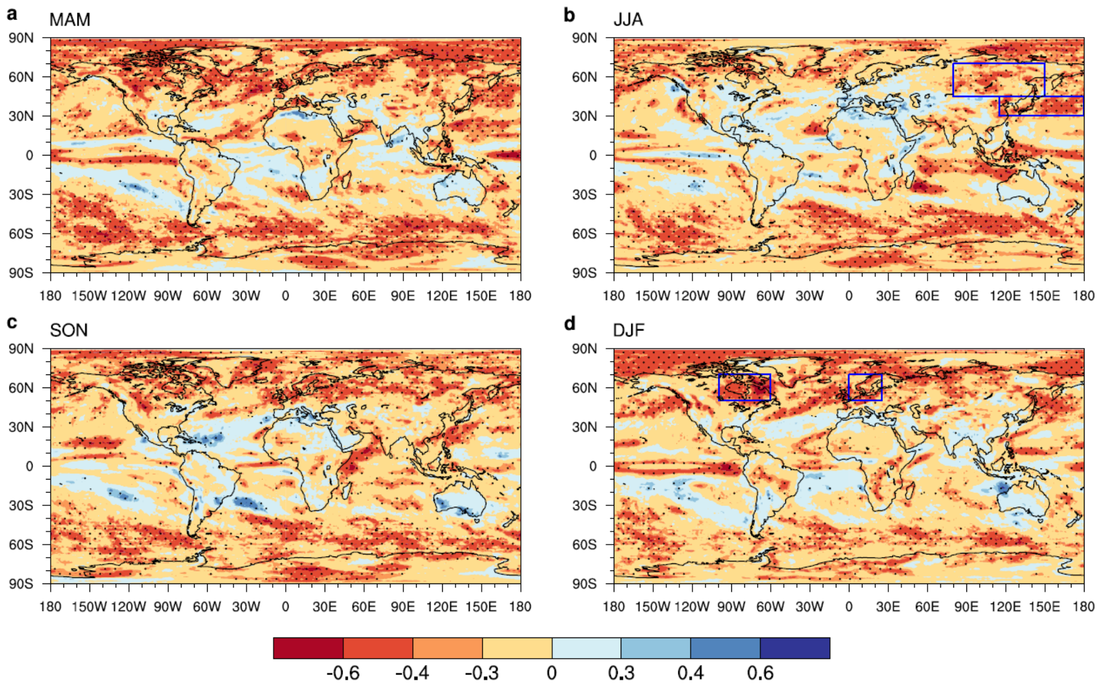

Supplementary Fig. 7 The emergent relationship in model simulations. Same as Fig. 1 in the main paper but for 1-day precipitation events (pr1d). Here extreme precipitation is defined as those exceeding the 95th percentile in the baseline (R95); precipitation variability is measured by the difference between the 95th and 50th percentile precipitation events (R95-R50).

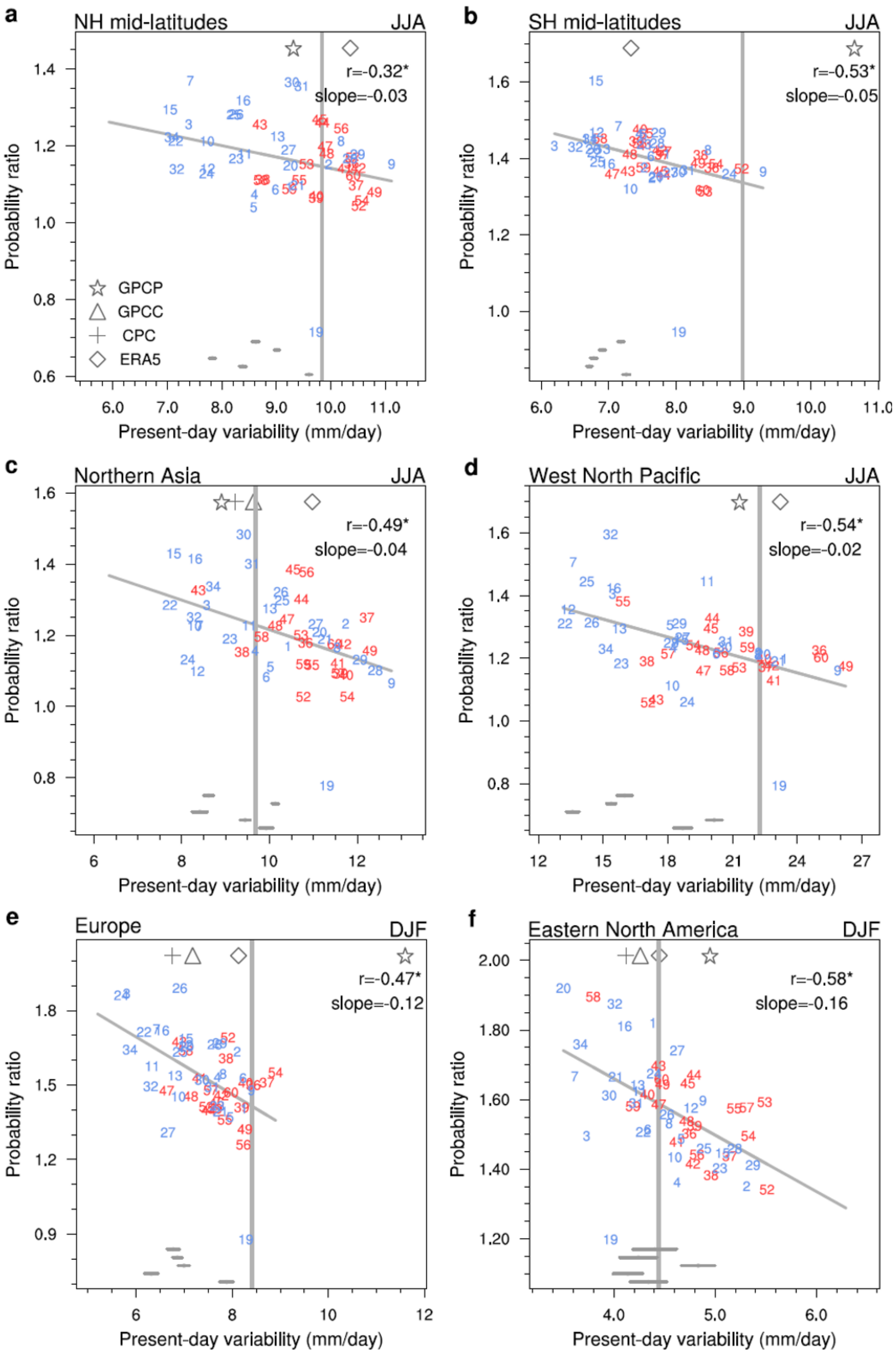

Supplementary Fig. 8 The emergent relationship over typical regions. Same as Fig. 2 in the main paper but for 1-day precipitation events (pr1d). Here extreme precipitation is defined as those exceeding the 95th percentile in the baseline (R95); precipitation variability is measured by the difference between the 95th and 50th percentile precipitation events (R95-R50).

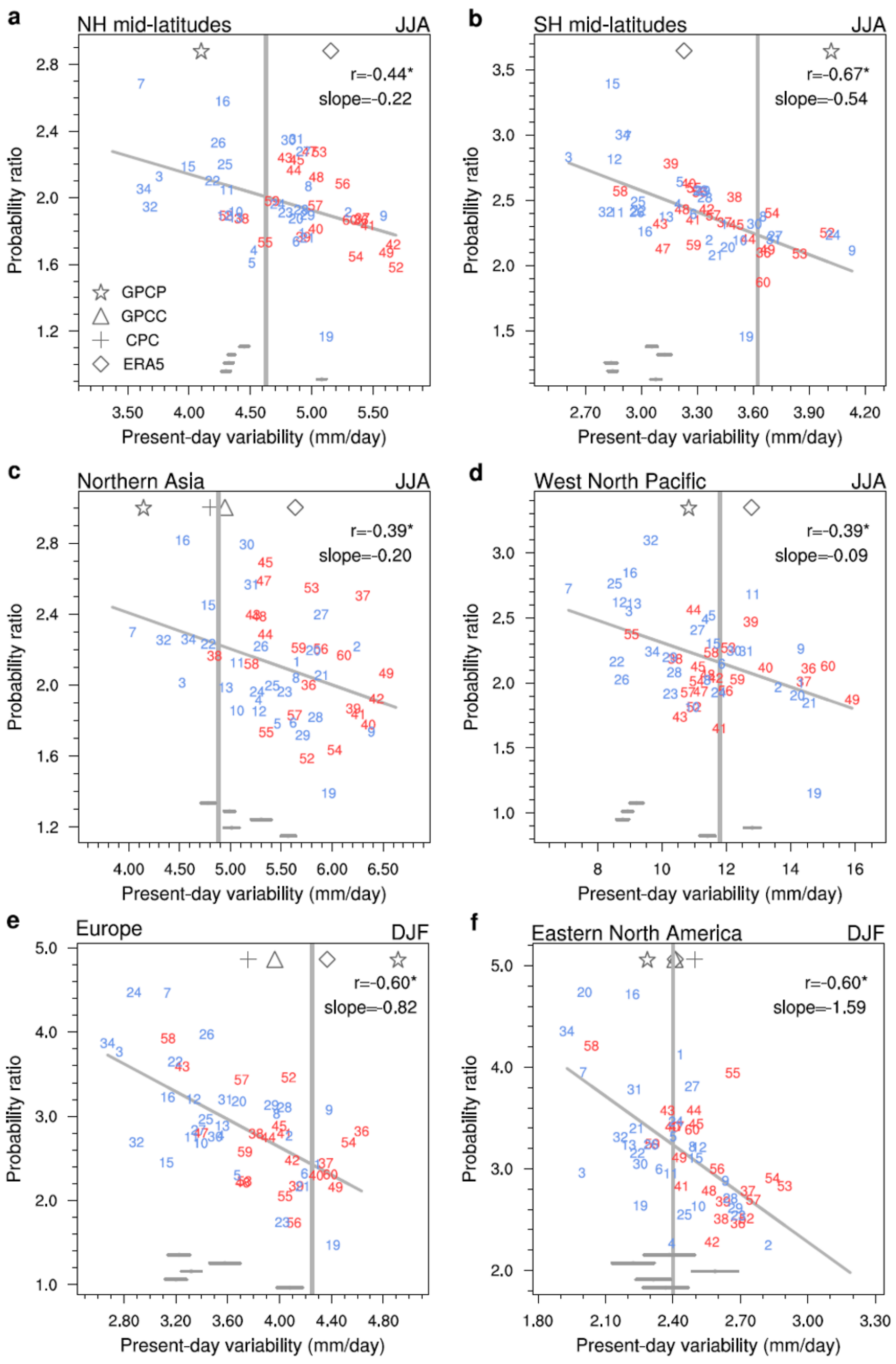

Supplementary Fig. 9 The emergent relationship over typical regions. Same as Fig. 2 in the main paper but extreme precipitation is defined as the 99th percentile events (R99). Here 5-day precipitation events (pr5d) are shown; precipitation variability is measured by the difference between the 95th and 50th percentile precipitation events (R95-R50).

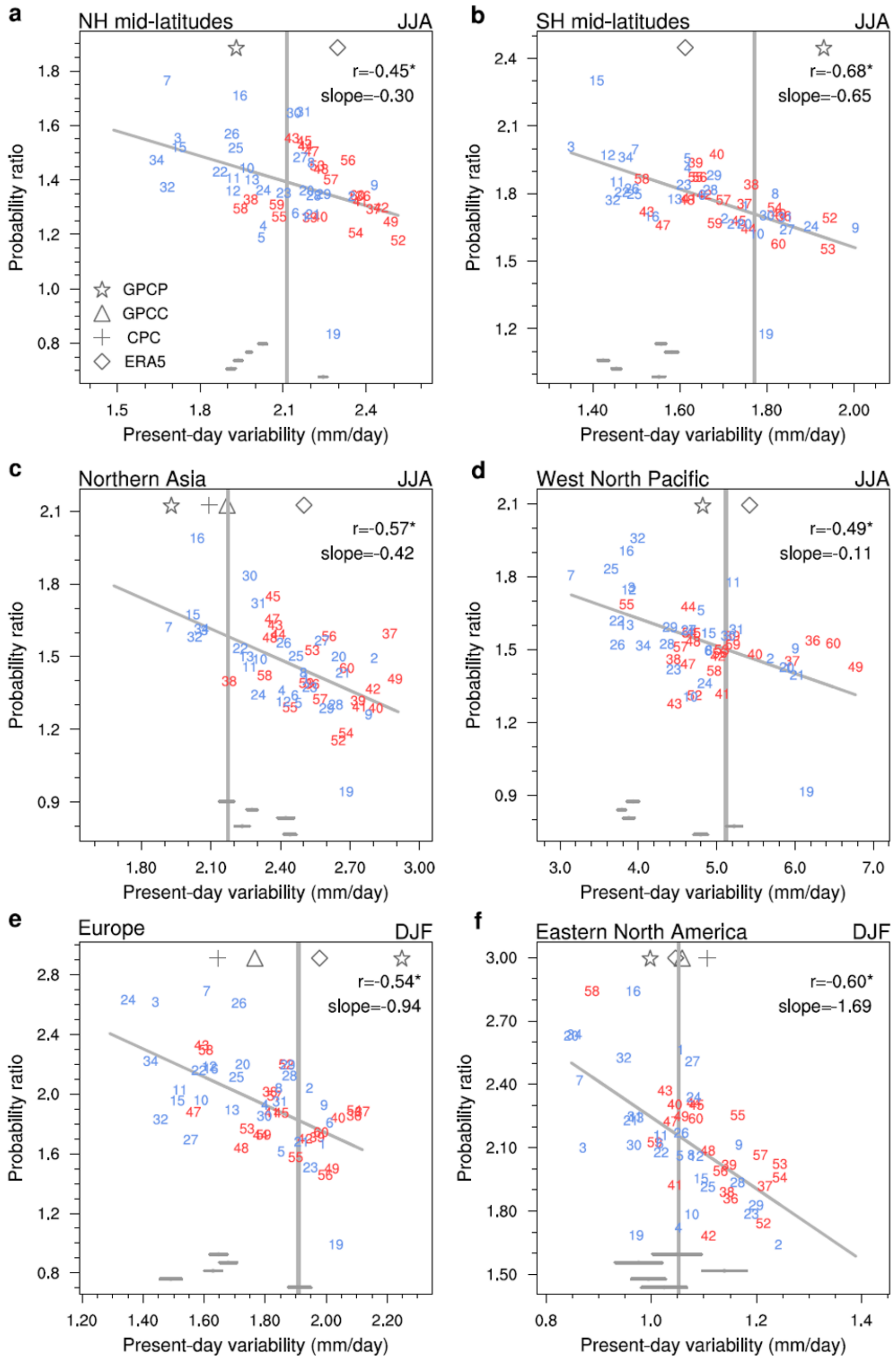

Supplementary Fig. 10 The emergent relationship over typical regions. Same as Fig. 2 in the main paper but precipitation variability is measured by the standard deviation of precipitation. Here 5-day precipitation events (pr5d) are shown; extreme precipitation is defined as the 95th percentile events (R95).

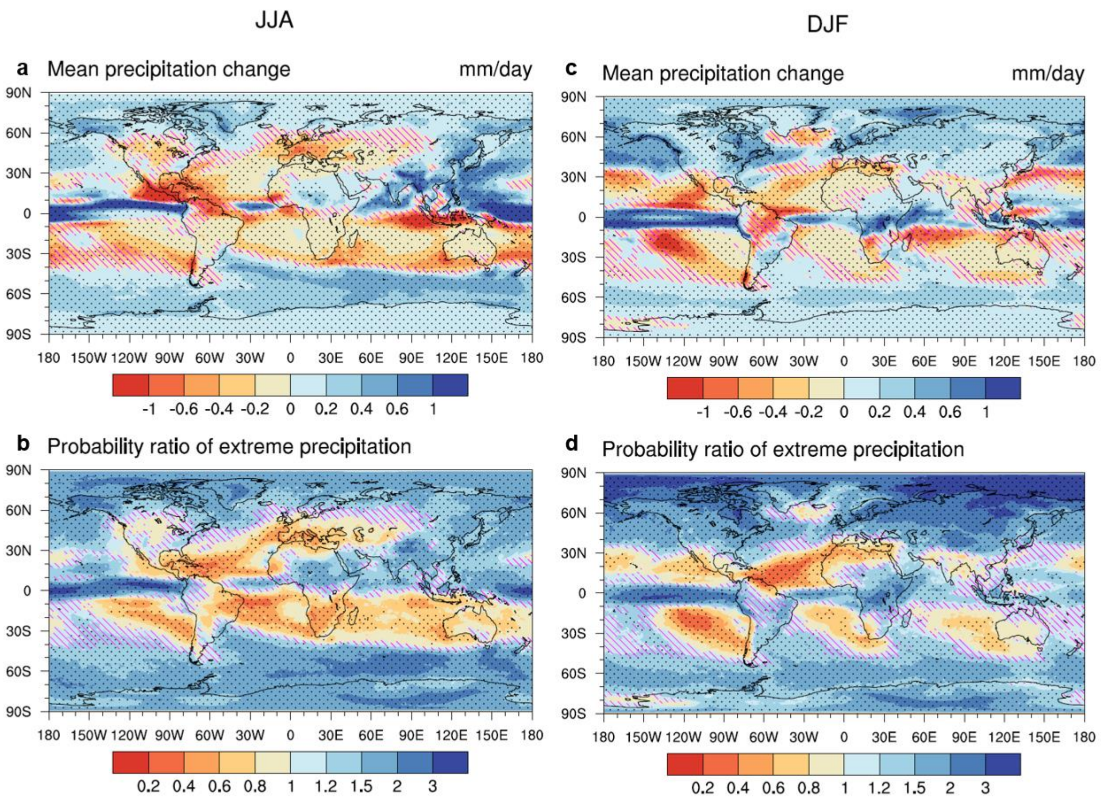

Supplementary Fig. 11 Mean and extreme precipitation changes. (a,c) Mean precipitation change (multi-model ensemble median across CMIP5 and CMIP6; unit: mm/day). **(b,d)** Probability ratio of extreme precipitation change (multi-model ensemble median; unit: 1). Only the results of JJA (June-to-August; **a-b**) and DJF (December-to-February; **c-d**) are shown for brevity. Dots indicate where at least 75% of models agree on the sign of change. Megenta hatching indicates where the multi-model median projects decreases in mean precipitation but increases in extreme precipitation. The patterns of change are insensitive to warming levels and extreme event definitions, thus the change between present day and a 3°C warming level is shown, and the 95th percentile for 5-day precipitation events (pr5d) is used to define extreme events.

Supplementary Table 1 CMIP5 models used in this study. The first realization in historical simulations and RCP8.5 projections in each model are used. The model numbers correspond to those in scatterplots. Underlines mark models that provide daily precipitation data from 1pctCO2 experiments.

Model No.	Model	Institute/Country	Resolution
1	ACCESS1.0	CSIRO–BOM/Australia	145 × 192
2	ACCESS1.3	CSIRO–BOM/Australia	145 × 192
3	BNU-ESM	BNU/China	64 × 128
4	CCSM4	NSF–DOE–NCAR/USA	192 × 288,
5	CESM1-BGC	NSF–DOE–NCAR/USA	192 × 288
6	CESM1-CAM5	NSF–DOE–NCAR/USA	192 × 288
7	CMCC-CESM	CMCC/Italy	48 × 96
8	CMCC-CMS	CMCC/Italy	96 × 192
9	CMCC-CM	CMCC/Italy	240 × 480
10	CNRM-CM5	CNRM–CERFACS/France	128 × 256
11	CSIRO-Mk3.6.0	CSIRO–QCCCE/Australia	96 × 192
12	CanESM2	CCCma/Canada	64 × 128
13	EC-EARTH	EC-Earth-Consortium/EU	160 × 320
14	FGOALS-g2	IAP-CAS/China	60 × 128
15	FGOALS-s2	IAP-CAS/China	108 × 128
16	GFDL-CM3	NOAA–GFDL/USA	90 × 144

17	GFDL-ESM2G	NOAA–GFDL/USA	90 × 144
18	GFDL-ESM2M	NOAA–GFDL/USA	90 × 144
19	HadGEM2-AO	MOHC/UK	145 × 192
20	HadGEM2-CC	MOHC/UK	145 × 192
21	HadGEM2-ES	MOHC/UK	145 × 192
22	IPSL-CM5A-LR	IPSL/France	96 × 96
23	IPSL-CM5A-MR	IPSL/France	143 × 144
24	IPSL-CM5B-LR	IPSL/France	96 × 96
25	MIROC-ESM-CHEM	MIROC/Japan	64 × 128
26	MIROC-ESM	MIROC/Japan	64 × 128
27	MIROC5	MIROC/Japan	128 × 256
28	MPI-ESM-LR	MPI-M/Germany	96 × 192
29	MPI-ESM-MR	MPI-M/Germany	96 × 192
30	MRI-CGCM3	MRI/Japan	160 × 320
31	MRI-ESM1	MRI/Japan	160 × 320
32	NorESM1-M	NCC-NMI/Norway	96 × 144
33	bcc-csm1-1-m	BCC–CMA/China	160 × 320
34	bcc-csm1-1	BCC–CMA/China	64 × 128
35	inmcm4	INM/Russia	120 × 180

Supplementary Table 2 CMIP6 models used in this study. The first realization in historical simulations and SSP5-8.5 projections in each model are used. The model numbers correspond to those in scatterplots. Underlines mark models that provide daily precipitation data from 1pctCO2 experiments.

**Model No.**	**Model**	**Institute/Country**	**Resolution**
36	ACCESS-CM2	CSIRO-BOM/Australia	145 × 192
37	ACCESS-ESM1-5	CSIRO-BOM/Australia	144 × 192
38	BCC-CSM2-MR	BCC-CMA/China	160 × 320
39	CESM2-WACCM	NCAR/USA	192 × 288
40	CESM2	NCAR/USA	192 × 288
41	CNRM-CM6-1	CNRM-CERFACS/France	128 × 256
42	CNRM-ESM2-1	CNRM-CERFACS/France	128 × 256
43	CanESM5	CCCma/Canada	64 × 128
44	EC-Earth3-Veg	EC-Earth-Consortium/EU	256 × 512
45	EC-Earth3	EC-Earth-Consortium/EU	256 × 512
46	FGOALS-g3	LASG-IAP/China	90 × 180
47	GFDL-CM4	GFDL-NOAA/USA	180 × 288

48	GFDL-ESM4	GFDL-NOAA/USA	180 × 288
49	HadGEM3-GC31-LL	MOHC/UK	144 × 192
50	INM-CM4-8	INM/Russia	120 × 180
51	INM-CM5-0	INM/Russia	120 × 180
52	IPSL-CM6A-LR	IPSL/France	143 × 144
53	MIROC6	MIROC/Japan	128 × 256
54	MPI-ESM1-2-HR	MPI-M/Germany	384 × 384
55	MPI-ESM1-2-LR	MPI-M/Germany	96 × 192
56	MRI-ESM2-0	MRI/Japan	160 × 320
57	NESM3	NUIST/China	96 × 192
58	NorESM2-LM	NCC-NMI/Norway	96 × 144
59	NorESM2-MM	NCC-NMI/Norway	192 × 288
60	UKESM1-0-LL	MOHC/UK	144 × 192

Supplementary Table 3 Five Single-Model Initial-condition Large Ensembles (SMILEs) providing daily precipitation data that are used in this study.

**Model**	**Institute/Country**	**Resolution**	**Number of Members**
CESM1	NCAR/USA	192 × 288	40
CanESM2	CCCma/Canada	64 × 128	50
CSIRO-MK3.6	CSIRO-QCCCE/Australia	96 × 192	30
GFDL-CM3	NOAA-GFDL/USA	90 × 144	20
EC-EARTH	EC-Earth-Consortium/EU	160 × 320	16
